# Prenatal Exposure to PBDEs and Neurodevelopment

**DOI:** 10.1289/ehp.0901340

**Published:** 2010-01-04

**Authors:** Julie B. Herbstman, Andreas Sjödin, Matthew Kurzon, Sally A. Lederman, Richard S. Jones, Virginia Rauh, Larry L. Needham, Deliang Tang, Megan Niedzwiecki, Richard Y. Wang, Frederica Perera

**Affiliations:** 1 Columbia Center for Children’s Environmental Health, Department of Environmental Health Sciences, Mailman School of Public Health, Columbia University, New York, New York, USA; 2 Division of Laboratory Sciences, National Center for Environmental Health, Centers for Disease Control and Prevention, Atlanta, Georgia, USA

**Keywords:** biomarkers, children, neurodevelopment, PBDEs, polybrominated diphenyl ethers, prenatal, World Trade Center, WTC

## Abstract

**Background:**

Polybrominated diphenyl ethers (PBDEs) are widely used flame retardant compounds that are persistent and bioaccumulative and therefore have become ubiquitous environment contaminants. Animal studies suggest that prenatal PBDE exposure may result in adverse neurodevelopmental effects.

**Objective:**

In a longitudinal cohort initiated after 11 September 2001, including 329 mothers who delivered in one of three hospitals in lower Manhattan, New York, we examined prenatal PBDE exposure and neurodevelopment when their children were 12–48 and 72 months of age.

**Methods:**

We analyzed 210 cord blood specimens for selected PBDE congeners and assessed neurodevelopmental effects in the children at 12–48 and 72 months of age; 118, 117, 114, 104, and 96 children with available cord PBDE measurements were assessed at 12, 24, 36, 48, and 72 months, respectively. We used multivariate regression analyses to evaluate the associations between concentrations of individual PBDE congeners and neurodevelopmental indices.

**Results:**

Median cord blood concentrations of PBDE congeners 47, 99, and 100 were 11.2, 3.2, and 1.4 ng/g lipid, respectively. After adjustment for potential confounders, children with higher concentrations of BDEs 47, 99, or 100 scored lower on tests of mental and physical development at 12–48 and 72 months. Associations were significant for 12-month Psychomotor Development Index (BDE-47), 24-month Mental Development Index (MDI) (BDE-47, 99, and 100), 36-month MDI (BDE-100), 48-month full-scale and verbal IQ (BDE-47, 99, and 100) and performance IQ (BDE-100), and 72-month performance IQ (BDE-100).

**Conclusions:**

This epidemiologic study demonstrates neurodevelopmental effects in relation to cord blood PBDE concentrations. Confirmation is needed in other longitudinal studies.

Polybrominated diphenyl ethers (PBDEs) are widely used flame retardant compounds applied to a wide array of textiles, building materials, and electronic equipment, including computers and televisions. Because they are additives rather than chemically bound to consumer products, they have the propensity to be released into the environment (Darnerud et al. 2001). PBDEs are persistent organic chemicals, and some congeners can bioaccumulate; therefore they have become ubiquitous contaminants detectable in the environment, in animals, and in humans (Hites 2004; Sjodin et al. 2008b).

A number of toxicologic studies have demonstrated that exposure to PBDEs may have endocrine-disrupting effects. Most of these studies have focused on thyroid hormone disruption and a smaller number on disruption of the estrogen/androgen hormone system [reviewed by Darnerud (2008)]. Endocrine disruption during critical developmental periods may result in irreversible effects on differentiating tissue, including the brain (Bigsby et al. 1999). Causal relationships between prenatal exposure to PBDEs and indices of developmental neurotoxicity have been observed in experimental animal models [reviewed by Costa and Giordano (2007)]. Thus, the disruption of endocrine pathways by prenatal exposure to hormonally active environmental chemicals may affect neurodevelopment in children.

Although the association between prenatal exposure to PBDEs and adverse neurodevelopmental effects has been observed in animal models, it has not been adequately explored in human populations. In a longitudinal cohort study initiated by the Columbia Center for Children’s Environmental Health (CCCEH), we examined the impact of prenatal exposures to selected toxicants, including PBDEs, that may be present in the ambient environment but may also have been emitted from the World Trade Center (WTC) buildings in New York City after the 11 September 2001 (9/11) terrorist attack. Here we report the relationship between prenatal PBDE and polybrominated biphenyl (PBB-153) measured in umbilical cord blood in humans and indicators of neurodevelopment at 12–48 and 72 months of age.

## Methods

### Study population

We established a prospective cohort study of women who were pregnant on 11 September 2001 and subsequently delivered at one of three downtown hospitals including Beth Israel, St. Vincent’s (and St. Vincent’s affiliated Elizabeth Seton Childbearing Center), which are all approximately 2 miles from the WTC site, and New York University Downtown Hospital, which is within a half-mile of the WTC site. The study methods have been described previously (Lederman et al. 2004). In brief, beginning 12 December 2001 [when institutional review board (IRB) approval was obtained], women were approached in the hospital when they presented for labor and delivery. The women were briefly screened for eligibility, recruited, and enrolled, and they consented before delivery. This study was conducted in accordance with all applicable requirements of the United States (including IRB approval), and all human participants gave written informed consent before participation in this study. Eligible women included those who were between 18 and 39 years of age, reported smoking < 1 cigarette per day during pregnancy, were pregnant on 11 September 2001 (based on their estimated date of conception), and reported no diabetes, hypertension, HIV infection or AIDS, or use of illegal drugs in the preceding year. Not all mothers agreed to have their child followed after birth. For example, some of the Chinese children were to be raised in China [see Supplemental Material, Table 1 (doi:10.1289/ehp.0901340) for follow-up information].

### Data collection

Medical records of the mother and newborn were abstracted for information relating to pregnancy, delivery, and birth outcomes. Interviews were conducted (generally the day after delivery) by bilingual interviewers in the preferred or native language (English, Spanish, or Chinese) of the participants. Demographic information, reproductive history, background environmental exposures, occupational history, and the location of the residences and workplaces of the woman during each of the 4 weeks after 11 September 2001 were determined during this interview. Maternal intelligence was measured using the Test of Non-Verbal Intelligence, Second Edition (TONI-2), a 15-min, language-free measure of general intelligence that is relatively stable and free of cultural bias (Brown et al. 1990).

### Developmental assessment

When the children were approximately 12, 24, and 36 months of age, the Bayley Scales of Infant Development, Second Edition (BSID-II) were administered, providing scores from the Mental Development Index (MDI) and the Psychomotor Development Index (PDI). The BSID-II is a widely used developmental test designed for children 12–42 months of age that is norm-referenced and can be used to identify children with developmental delay. The assessment provides a developmental quotient (raw score/chronological age), generating a continuous MDI or PDI score, both with mean ± SD = 100 ± 15.

When the children were 48 and 72 months of age, the Wechsler Preschool and Primary Scale of Intelligence, Revised Edition (WPPSI-R) was administered, which measures cognitive development and contains verbal and nonverbal performance tests. We used the WPPSI-R because of its availability in Chinese, rather than the third edition.

Not all children were available for all developmental assessments, resulting in different numbers of children tested at each age. Assessments were conducted in the first language of the child (English or Chinese) by trained research technicians. In some cases, when the primary language of the child was not English or Chinese (e.g., Yiddish), we relied on maternal translation. Statistical analyses for this study were conducted with and without the children for whom the child’s primary language was not English or Chinese (*n* = 30), and the results were similar (data not shown).

Most assessments were conducted at the CCCEH. However, a proportion of the assessments were conducted in the child’s home if the parents were unable or unwilling to come to the center to complete the follow-up [see Supplemental Material, Table 1 (doi:10.1289/ehp.0901340) for details].

### Blood collection

Umbilical cord blood was collected at delivery, and maternal blood was typically collected on the day after delivery. On average, 30.7 mL blood was collected from the umbilical cord, and 30–35 mL blood was collected from the mothers. Samples were transported to the laboratory and processed within several hours of collection. The buffy coat, packed red blood cells, and plasma were separated and stored at −70°C. Frozen plasma from 210 cord samples was transferred on dry ice to the Centers for Disease Control and Prevention for laboratory analyses for the PBDEs and PBB-153. The concentrations of these chemicals in the cord blood were used as an indicator of fetal exposure during gestation (Mazdai et al. 2003; Qiu et al. 2009).

### Laboratory methods

Details regarding the analysis of the plasma samples for PBDEs are given elsewhere (Hovander et al. 2000; Sjodin et al. 2004). Briefly, the samples were automatically fortified with ^13^C-labeled internal standards. The samples were subjected to an initial liquid/liquid extraction with hexane:methyl-*tert*-butyl ether after denaturation with 1 M HCl and isopropanol (Hovander et al. 2000). Thereafter, coextracted lipids were removed on a silica:silica/sulfuric acid column using the Rapid Trace equipment (Zymark, Hopkinton, MA) for automation. Final determination of the target analytes was performed by gas chromatography-isotope dilution high-resolution mass spectrometry employing an MAT95XP (ThermoFinnigan MAT, Bremen, Germany) instrument (Sjodin et al. 2004). Concentrations of target analytes were reported as picograms per gram whole weight (weight of plasma) and nanograms per gram lipid weight (weight of plasma lipids). The plasma lipid concentrations were determined using commercially available test kits from Roche Diagnostics Corp. (Indianapolis, IN) for the quantitative determination of total triglycerides (product no. 011002803-0600) and total cholesterol (product no. 011573303-0600). Final determinations were made on a Hitachi 912 Chemistry Analyzer (Hitachi, Tokyo, Japan). Limits of quantification were determined in relation to the method blanks and in relation to the quantification limit of the instrument, which is proportional to the sample size. Cotinine concentrations were measured in cord and maternal blood by use of liquid chromatography in conjunction with atmospheric pressure ionization tandem mass spectrometry (Bernert et al. 1997).

The plasma samples were analyzed for the following PBDE congeners (by International Union of Pure and Applied Chemistry numbers): 2,2,2′,4,4′-tetraBDE (BDE-47); 2,2′,3,4,4′-pentaBDE (BDE-85); 2,2′,4,4′,5-pentaBDE (BDE-99); 2,2′,4,4′,6-pentaBDE (BDE-100); 2,2′,4,4′,5,5′-hexaBDE (BDE-153); 2,2′,4,4′,5,6′-hexaBDE (BDE-154); 2,2′,3,4,4′,5′,6-heptaBDE (BDE-183); and 2,2′,4,4′,5,5′-hexaBB (BB-153).

### Quality control/quality assurance

We determined background levels by measuring the level of target analytes in blank samples in the same run as the study samples (three blanks per 24 study samples). All concentrations reported were corrected for the average amount present in the blank samples. The limit of detection (LOD) when no analytical background was detected in blank samples was defined as a signal-to-noise ratio > 3. When an analytical background was detected in the blanks, the LOD was defined as three times the SD of the blanks.

The plasma samples used in this cohort were not collected solely for the purpose of PBDE analysis. Therefore, we examined the ratio of BDE-99 over BDE-47 for any indication of contamination from indoor particulate matter, with the assumption that a high ratio would indicate sample contamination during sample collection. The median ratio of BDE-99 over BDE-47 is 1.2 in residential dust samples (Sjodin et al. 2008a), whereas in human samples this ratio is typically significantly lower. In the 2003–2004 National Health and Nutrition Examination Survey (NHANES), the median ratio of BDE-99 to BDE-47 was 0.23, whereas the 95th percentile of this ratio was 0.43 (Sjodin et al. 2008b). In our study, we found that 16 of 210 samples had a BDE-99 to BDE-47 ratio > 0.43, corresponding to 7.6% of the samples. This frequency of samples having a ratio > 0.43 is similar to that of the NHANES survey, and we can thus conclude that no detectable contamination occurred during the collection of the cord samples in this study. Statistical analyses for this study were conducted both including and excluding the aberrant samples (*n* = 16), and the results were similar (data not shown).

### WTC exposure

In previous analyses in this cohort, we used two indices to describe exposure to the WTC: geographic proximity to the WTC during the first month after 9/11 and timing of exposure relative to date of delivery. We found that women who lived closest to the WTC during the first month after 9/11 (constituting the group we would estimate to have the largest exposure to the WTC) did not have higher concentrations of PBDEs compared with those who lived farther from the towers in the first 4 weeks after the attack. However, we found that women who delivered sooner after 9/11 (constituting the group who were further along in their pregnancy on 9/11) tended to have higher cord blood concentrations of PBDEs (unpublished data). Because we quantify prenatal PBDE exposure using a biological marker that integrates exposure from all sources, the source of the PBDEs is not relevant to the effect of prenatal exposure to PBDEs on neurodevelopment. To our knowledge, PBDEs are not associated with any other neurotoxic exposure that could confound the observed associations.

### Statistical methods

Concentrations of PBDEs were lipid- and natural log-adjusted. PBDEs commonly detected in cord blood (detected in > 55% of samples) were handled as continuous variables in the statistical models. This was the case for BDEs 47, 99, 100, and 153. We used the LOD divided by the square root of 2 for concentrations below the LOD. Based on the log-normal distribution of each of these BDE congeners, we also compared participants having cord concentrations in the highest 20% with those in the lowest 80% of the population distribution to evaluate the impact of having exposures at the high end of the exposure distribution. This categorization was selected because it distinguished those with exposures in the tail of the log-normal distribution. The majority of cord samples (> 50%) had levels below the limits of detection for BDEs 85, 154, and 183, and BB-153. We evaluated BDE-85 and BB-153 as dichotomous measures (detected vs. nondetected); we did not analyze BDEs 154 and 183 because only 6% and 4% of the samples, respectively, had detectable concentrations (Table 1).

We generated descriptive statistics and evaluated bivariate associations using analysis of variance and chi-square tests to compare stratum-specific means and proportions, respectively. We examined the data using lowess curves and determined that linear models using natural logarithmic (ln)-transformed PBDE concentrations fit the data well. Therefore, we conducted multivariate linear regression analyses to evaluate the relationships between prenatal PBDE concentrations (using continuous measures for ln-transformed BDEs 47, 99, 100, and 153 and dichotomous measures for BDEs 85, 154, and 183) and continuous scores on developmental tests (MDI and PDI at 12, 24, and 36 months and Full, Verbal, and Performance scores at 48 and 72 months). We were not able to consider developmental test scores as dichotomous measures, using the test-specific recommended cutoffs for defining children as “delayed” or “borderline delayed” because of the small sample size and small number of children who met these criteria.

We selected covariates for inclusion in multivariate models based on their *a priori* association with neurodevelopment (Tong and Lu 2001), including age at testing, sex of child, ethnicity (Asian, black, white, or other), environmental tobacco smoke (ETS) exposure in the home [yes/no; based on self-report and validated in this data set by cotinine measured in cord blood, using methodology described by Jedrychowski et al. (2009)] and IQ of the mother. We also considered the inclusion of additional covariates if they changed the beta coefficient for PBDEs > 10% when they were added to the *a priori* set one at a time. This resulted in a final covariate set that added to our *a priori* set gestational age at birth (based on the best obstetric estimate), maternal age, maternal education, material hardship during pregnancy (defined as having gone without either food, shelter, gas/electric, clothing, or medication/medical care because of financial constraint), and breast-feeding [considering both breast-feeding duration and exclusiveness, defined by Lederman et al. (2008)]. The results of these models are presented as model 1. We also created a model 2, which included all the previous covariates plus two study-specific variables: the language (including whether the mother aided in translation) and location (home or study site) of the interview and assessment. We explored the effects of whether the mother ate fish/seafood when she was pregnant (yes/no) and also the effects of cord blood total mercury and lead concentrations (continuous measures), but found that these covariates did not materially change the relationships between PBDE concentrations and developmental indicators.

To determine whether only a few cases could have a substantial association with the adjusted PBDE regression coefficients, we used AV plots to examine the residuals from the regression lines for the adjusted PBDE regression coefficients to find possible influential cases. These are cases that are outliers both for the outcomes (PDI, MDI, WPPSI) and the independent variables of interest (PBDE compounds). When the possible influential cases were removed, the largest changes in the regression coefficients were < 1 point, with no changes in significance levels, using *p* < 0.05 as a cut point.

## Results

Median cord concentrations of PBDE congeners 47, 99, and 100 in the full cohort were 11.2, 3.2, and 1.4 ng/g lipid; 81.4%, 59.5%, and 63.6%, respectively, were above the LOD (Table 1). Overall concentrations and the proportion of participants with PBDE concentrations above the LOD were not significantly different in the study subsample. The proportion of participants with detectable concentrations of BDEs 85, 153, 154, and 183 ranged from approximately 4% to 50%. PBDE congeners 47, 99, and 100 were highly intercorrelated (*r* = 0.74–0.88).

Characteristics of the full cohort (*n* = 329), the subset of 210 participants with cord PBDE (and PBB) measurements, and the subset of 152 with both cord measurement and a neurodevelopmental test are shown in Table 2. Those with cord blood measurements were similar to the full cohort except that there were proportionally more Chinese participants with cord blood measurements (34.3% compared with 28.0% in the full cohort) and those with measurements were slightly smaller in birth length. The study sample and the full cohort were similar except that mothers included in the study sample were slightly older at the time of delivery (31.2 vs. 30.2 years, *p* < 0.01) and were more educated (not statistically significant). Those in the study sample were more likely to have completed maternal IQ measurements, which is expected, considering that this measurement was collected at follow-up visits, not at the delivery hospital. There were no differences in the proportions working and/or living closest to the WTC (within 1 or 2 miles) at the time of the attack.

There were 118, 117, 114, 104, and 96 children with available cord PBDE measurements who also had a developmental assessment at 12, 24, 36, 48, and 72 months of age, respectively. For those assessed at all of these time points, the median cord plasma concentrations of BDE-47 and 99 were 12.1 ng/g lipid and 3.5 ng/g lipid. For BDE-100, median concentrations were approximately 1.5 ng/g lipid for children assessed at 12–48 months; for those assessed at 72 months, the median cord blood concentration was 1.4 ng/lipid.

In cross-sectional analyses using multivariate linear regression, prenatal exposure to BDE-47 was negatively associated with neurodevelopmental indices (Table 3). These relationships were statistically significant for 12-month PDI (borderline), 24-month MDI, and 48-month Full and Verbal IQ scores. For every ln-unit change in BDE-47, scores were, on average, 2.1–3.1 points lower on developmental indices. For BDE-99 (Table 4), statistically significant negative associations were detected for 24-month MDI [β = −2.82; 95% confidence interval (CI), −4.86 to −0.78]. Prenatal exposure to BDE-100 was negatively associated with neurodevelopmental indices (Table 5), with statistically significant relationships observed for 24-month MDI, 48-month Full, Verbal, and Performance IQ scores, and 72-month Performance IQ scores. For every ln-unit change in BDE-100, scores were, on average, 3.4–4.0 points lower on developmental indices. For BDE-153 (Table 6), statistically significant negative associations were detected for 48-month and 72-month Full and Performance IQ scores. For every ln-unit change in BDE-153, scores were, on average, 3.1–4.2 points lower. The strength of association between BDE-153 and IQ scores was much larger in the adjusted models compared with the univariate model. It appears that the strong positive association of maternal education between IQs at ages 48 and 72 months was responsible for much of this change.

**Figure 1:**
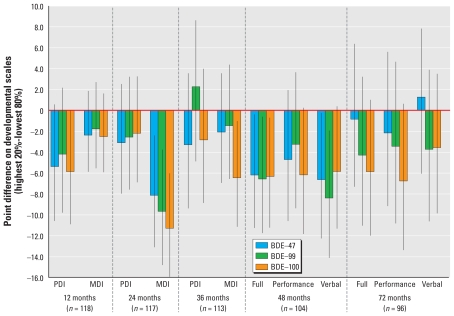
Difference in mean developmental score (and 95% confidence interval around the mean) comparing individuals in the highest quintile (20%) of exposure with those in the lower 80% of BDEs 47, 99, and 100. Mean differences were adjusted for age at testing, race/ethnicity, IQ of mother, sex of child, gestational age at birth, maternal age, ETS (yes/no), maternal education, material hardship, breast-feeding, language, and location of interview.

We also evaluated the difference in mean developmental score comparing children who were in the highest 20% of the prenatal exposure distribution with those in the lower 80% of the distribution for BDEs 47, 99, and 100 (Figure 1). We found that, on average, children with the higher prenatal concentrations of BDEs 47, 99, and 100 scored lower than the rest of the population on nearly all neurodevelopmental indices at all time points (12–48 and 72 months). These differences ranged in magnitude; the largest differences were observed with all three congeners for the 24-month MDI (statistically significant differences of −7.7, −9.3, and −10.9 points for BDEs 47, 99, and 100, respectively) and for 48-month Verbal and Full IQ scores (ranging from −5.5 to −8.0 points). For BDE-153, adjusting for the same covariate set, those in the highest 20% of the exposure distribution scored, on average, 6.3 points lower at 48 months (95% CI, −13.0 to 0.4) and 8.1 points lower at 72 months (95% CI, −15.6 to −0.6) on the performance IQ scale.

We used multivariate linear regression models to evaluate whether having detectable prenatal concentrations of BDE-85 and BB-153 was significantly related to developmental indices. Adjusting for the exact age of the child at test administration, ethnicity, IQ of the mother, sex of the child, gestational age at birth, maternal age, ETS (yes/no), maternal education, material hardship, and breast-feeding, we found that those with detectable cord concentrations of BDE-85 scored, on average, 11 points lower on the 24-month MDI (95% CI, −17.0 to −5.2); 6.4 points lower on 24-month PDI (95% CI, −11.8 to −0.8); 7.7 points lower on 36-month PDI (95% CI, −15.0 to −0.4); 6.5 points lower on 48-month Verbal IQ (95% CI, −13.3 to 0.2); and 6.9 points lower on 48-month Full IQ (95% CI, −12.8 to −0.9). There were no statistically significant associations between prenatal BB-153 levels with developmental indices measured at any other ages.

## Discussion

We found evidence suggesting that children who had higher cord blood concentrations of BDEs 47, 99, and 100 scored lower on tests of mental and physical development at ages 12–48 and 72 months. These associations were significant for 12-month PDI (BDE-47); 24-month MDI (BDEs 47, 99, and 100); 48-month Full IQ (BDEs 47, 100, and 153); Verbal IQ (BDEs 47 and 100) and Performance IQ (BDEs 100 and 153); and 72-month full and Performance IQ (BDEs 100 and 153). Children who were in the highest 20% of cord blood concentrations of BDEs 47, 99, or 100 had significantly lower developmental scores compared with children who were in the lower 80% of the exposure distributions for these chemicals. These differences were particularly evident at 48 months of age.

Adverse neurodevelopmental effects associated with prenatal PBDE exposure can be detected both at early ages (12–36 months) and as the children age (48 and 72 months). Neurodevelopmental deficits documented by the WPPSI during the preschool period are an important predictor of subsequent academic performance (Kaplan 1993). Documenting the first appearance of potentially longer-term adverse effects at early ages is also important, because these indicators may identify children who could benefit from early intervention programs. The identification of later deficits may indicate the persistence of early effects and/or an increase in the magnitude of effect with age, as has been shown in some animal studies (e.g., Viberg et al. 2003a).

Our results are consistent across congeners and over time. This may be predictable because the PBDE congeners are highly correlated, and for individuals, repeated developmental scores are also correlated. Although the number of participants lost to follow-up between 12 and 72 months was relatively low (81% of subjects available for analyses at 12 months were also assessed at 72 months) and losses are independent of exposure, our overall sample is relatively small. Therefore, even small losses to follow-up may limit our power to detect significant differences in multivariate models. The resulting small sample size precluded the analysis of exposure effects on developmental delay, and we were unable to look at interactions. However, the developmental deficits of the magnitude we observed in this study are likely to have the largest functional impact on those who score at the lower end of the population distribution.

The only other epidemiologic study reporting the neurodevelopmental effects of prenatal exposure to PBDEs was published recently (Roze et al. 2009). In this study of 62 Dutch children, the authors present correlations between exposure to PBDEs (measured during the 35th week of pregnancy) and > 20 indices of child development and behavior at age 60–72 months. The authors report that prenatal PBDE exposure was associated with some adverse effects on development (reduced fine manipulative abilities and increased attentional deficits) as well as some beneficial effects (better coordination, better visual perception, and better behavior). The authors evaluated, but did not find statistically significant, correlations between prenatal PBDE exposures and any of the WPPSI-R domains. Our results are not consistent with these findings. However, important differences in exposure (median exposure in our population was 4 times higher for BDEs 47 and 99; 2.3 times higher for BDE-100; and one-fifth their concentration for BDE-153), sample size, and statistical analyses performed may account for some of the observed inconsistencies.

Our results are consistent with published toxicologic experiments [reviewed by Costa and Giordano (2007)]. For example, studies evaluating the neurodevelopmental effects of neonatal exposure to PBDEs in mice during critical developmental periods have reported altered habituation patterns (Viberg et al. 2003a, 2003b), hyperactivity (Gee and Moser 2008), and learning and memory deficits (Dufault et al. 2005; Viberg et al. 2003a). There is some evidence suggesting that BDE-99 is more potent than BDE-47 (Viberg et al. 2003a) and also that effects worsen (or are more apparent) with age (Viberg et al. 2003a). In general, we observed the largest associations with prenatal exposure to BDE-100, and the associations with prenatal exposures were still apparent, albeit not consistently significant, in our smaller sample examined at 72 months of age.

A number of potential mechanisms have been proposed to explain the cognitive and locomotive deficits observed in animals after PBDE exposure during critical developmental periods, including direct neurotoxic effects on neuronal and glial cells (Costa et al. 2008) resulting from changes in the quantity of cholinergic nicotinic receptors in the hippocampus (Viberg et al. 2003a) and induction of apoptotic cerebellar granule cell death (Reistad et al. 2006). In addition, there is compelling experimental and epidemiologic evidence suggesting that PBDEs can interfere with thyroid hormone pathways (Legler 2008). Because thyroid hormones are critical for normal brain development, this provides an attractive explanation for observed neurodevelopmental effects after neonatal PBDE exposure (Bigsby et al. 1999; Porterfield 2000). Toxicologic evidence corroborating this theory includes potentially causal associations between neonatal exposure to BDE-47, BDE-99, or commercial PBDE mixtures (DE-71 and Bromokal 70-5 DE) and reduced thyroxine (T_4_) concentrations in experimental murine models (Fowles et al. 1994; Hallgren et al. 2001; Kuriyama et al. 2007).

Although only limited human epidemiologic data are available, increased levels of BDEs 47, 99, and 100 in dust in the homes of adult human males recruited through a U.S. infertility clinic were associated with altered hormone levels. PBDEs were inversely associated with free androgen index and with luteinizing and follicle-stimulating hormones and were positively associated with inhibin B, sex hormone–binding globulin, and free T_4_ (Meeker et al. 2009). In another study of adult males, increased serum concentrations of PBDEs were positively related to T_4_ and inversely related to total triiodothyronine (T_3_) and thyroid-stimulating hormone (TSH) (Turyk et al. 2008). The positive associations between PBDEs and T_4_ levels demonstrated in these human studies are not consistent with the results from experimental animal models, raising the possibility that the underlying mechanism of the effect of PBDEs on thyroid disruption may differ among species. However, it is difficult to extrapolate findings from studies evaluating exposure effects in adults to prenatal exposures, because PBDEs may exhibit differential effects on thyroid hormone levels at different stages of the life span. A recent study of PBDEs measured in human cord blood of infants born to a cross-section of women delivering in Baltimore, Maryland, showed a consistent nonsignificant negative association with both total and free T_4_ in infants (Herbstman et al. 2008). More research is necessary to fully characterize the association of human prenatal exposure to PBDEs with thyroid hormone levels.

The exact mechanism of thyroid disruption by PBDEs in humans has not yet been elucidated, but two potential pathways through which PBDE exposure could lead to thyroid disruption have been proposed [reviewed by Zhang et al. (2008)]. The structural similarities of T_4_ and T_3_ to polyhalogenated aromatic hydrocarbons suggest that hydroxylated PBDE metabolites could displace thyroid hormones from thyroid transport proteins (i.e., transthyretin), altering free thyroid hormone levels (Turyk et al. 2008). Alternatively (or in addition), PBDEs might affect hormone levels by influencing thyroid hormone synthesis and/or stimulating thyroid hormone metabolism (Szabo et al. 2009; Turyk et al. 2008). Brain development in the fetus is contingent on the precise timing of thyroid hormone levels, particularly for T_4_, and deviations above or below the normal levels can lead to developmental deficits (Williams 2008). The fetus originally derives all thyroid hormone from the mother, but over the course of the pregnancy, its thyroid gland develops, and hormones produced within the fetus gradually replace the maternal source. The surge in maternal T_4_ in the first trimester, coupled with TSH inhibition, is thought to provide a supply of hormone during this critical developmental period, and alteration of T_4_ levels by PBDEs at this time could alter neurodevelopment (Williams 2008). Although low serum T_4_ from maternal hypothyroidism during gestation (e.g., iodine deficiency) is known to cause mental retardation in children, elevated levels of T_4_ have been associated with increased rates of miscarriage (Anselmo et al. 2004) and could potentially be linked to neurodevelopmental problems.

Because of their similar chemical structures, PBDEs and polychlorinated biphenyls (PCBs) have been compared in terms of their potential health effects. Although PCBs were banned in most industrialized countries > 25 years ago, they are still measurable in human and environmental samples because of their long half-lives in the environment and in humans (Talsness 2008). Prenatal exposure to PCBs has been shown in several cohort studies to significantly reduce cognitive function during childhood [reviewed by Schantz et al. (2003)] and has also been associated in some studies with altered thyroid hormone levels (Chevrier et al. 2007; Herbstman et al. 2008). Because of the structural similarity of PCBs and PBDEs, it has been postulated that they exert biological effects through similar processes.

This study population is unique in that participants were initially recruited to measure the extent and the effects of prenatal exposure to contaminants (including PBDEs) that were potentially released by the destruction of the WTC towers. Studies examining environmental samples collected pre- and post-9/11 near the WTC site found indications of higher concentrations of PBDEs after the attacks (Litten et al. 2003) and nearer to the WTC disaster site (Butt et al. 2004). These trends may be attributable to debris containing office equipment known to be treated with PBDEs (de Wit 2002). In our study population, cord plasma levels of PBDEs were not significantly related to residential distance from the WTC site. There is some evidence suggesting that PBDE exposure may be related to the WTC attack based on the gestational age on 9/11, such that women who were in the second half of their pregnancy on 9/11 had children with higher cord concentrations of PBDEs (unpublished data). It is also possible that just after 9/11, some women had elevated levels of PBDEs but that these levels declined with the passage of time between the peak exposure and delivery, resulting in lower observed levels. In either scenario, it is not clear how much this apparent association between gestational age on 9/11 and exposure concentration contributes to the body burden, and it is certain that sources other than the WTC are also accountable. In this report, our interest is in the association between the integrated prenatal PBDE exposure from multiple sources and neurodevelopment. It is also possible that there are other unknown factors associated with PBDEs that may confound the observed relationships between prenatal PBDE exposure and adverse neurodevelopment.

Levels of cord blood PBDEs in our population are consistent with those reported in other U.S. populations (Herbstman et al. 2007; Mazdai et al. 2003; Wu et al. 2007). Compared with cord blood measurements in an inner-city population in Baltimore, Maryland, our study population had slightly lower median concentrations (i.e., 11.2 ng/g lipid vs. 13.6 ng/g lipid for BDE-47) (Herbstman et al. 2007). In the Baltimore cohort as well as in this New York City cohort, higher cord PBDE concentrations were associated with mothers’ African American or non-Asian race/ethnicity, although a higher proportion of the Baltimore population was African American (70% vs. 15%) and a lower proportion was Asian (8% vs. 30%). Increasing maternal age was associated with lower PBDE concentrations in the Baltimore cohort but not in New York City; however the median maternal age was also lower in Baltimore (25 years vs. 30 years) (Herbstman et al. 2007). The demographic differences between these two populations may explain the small differences in blood levels observed at the population level.

Although dietary ingestion was once thought to be the largest route of PBDE exposure in humans, the similarity of PBDE levels in foods in Europe, Asia, and North America fails to adequately explain the high blood levels in the U.S. population (Frederiksen et al. 2009). Dust inhalation may be a more important exposure route to PBDEs, particularly BDEs 47, 99, and 100. In a review of median PBDE levels in dust and air samples, measured BDE-47 dust levels in Europe and North America were 32 and 429 ng/g of dust, respectively. Similar disparities were observed for BDE-99 and BDE-100 levels (Frederiksen et al. 2009; Sjodin et al. 2008a). Particular attention should be given to this exposure route in young children, who are more likely to encounter dust because of their proximity to the floor. Dust is estimated to contribute from 80 to 93% of PBDE exposure in toddlers, and their small body size compounds the effect of their exposures (Costa and Giordano 2007). In this study, we were not able to control for postnatal dust exposure.

In the general population, infants and toddlers have the highest body burden of PBDEs, and along with dust exposure, exposure via breast milk is thought to be a major contributor to this burden (Costa et al. 2008; Toms et al. 2009). Breast-fed infants are estimated to be exposed to 306 ng/kg body weight/day PDBE compared with 1 ng/kg body weight/day in adults, with the most prominent congeners being BDEs 47, 99, and 153 (Costa et al. 2008). In our study, breast-feeding rates were higher in children with higher cord PBDE levels, indicating that PBDEs measured in cord blood may underestimate the exposure of breast-fed children. Breast-feeding was, as expected, associated with higher scores on neurodevelopmental indices, making it an important potential confounder to include in multivariate statistical models.

## Conclusions

This report is among the first epidemiologic studies to demonstrate inverse associations between elevated cord blood concentrations of PBDEs and adverse neurodevelopmental test scores. These findings indicate a need for additional work to advance our understanding of the effects of perinatal exposure to PBDEs on neurodevelopment and to evaluate the role of thyroid hormones in this process. Additional PBDE congeners not measured in our study should also be examined to determine whether other congeners, including those that are highly brominated, play a role in developmental outcomes. Future work should also explore the possibility of interactions of PBDEs with other chemicals such as PCBs and dichlorodiphenyldichloroethylene. Although additional studies exploring the associations between PBDE exposure and developmental effects are underway, the identification of opportunities to reduce exposure to these compounds should be a priority.

## Figures and Tables

**Table 1:** Concentrations (ng/g lipid) of PBDEs and BB-153 in cord blood.

	Cord blood measurements (*n* = 210)				Cord measurements with > 1neurodevelopmental test (*n* = 152)			
	*n*	% > LOD	Median	Maximum	*n*	% > LOD	Median	Maximum
BDE-47	210	81.4	11.2	613.1	152	83.6	11.2	613.1
BDE-85	189	18.5	0.7	16.6	141	17.7	0.7	16.6
BDE-99	210	59.5	3.2	202.8	152	57.9	3.2	202.8
BDE-100	209	63.6	1.4	71.9	152	69.1	1.4	71.9
BDE-153	201	49.8	0.7	28.9	143	55.9	0.7	28.9
BDE-154	200	6.0	0.6	11.1	146	6.2	0.6	11.1
BDE-183	204	3.9	0.6	2.8	147	4.1	0.6	2.8
BB-153	197	11.2	0.6	8.0	145	13.1	0.9	8.0

**Table 2:** Characteristics of all cohort members (*n* = 329), participants with cord blood measurement of PBDEs (*n* = 210), and those included in our study sample (*n* = 152).

	All participants (*n* = 329)	Cord PBDEs (*n* = 210)	Cord measurements > 1 neurodevelopmental test (*n* = 152)
Maternal characteristics			
Maternal age (years)	30.2 ± 5.2	30.4 ± 5.1	31.2 ± 4.9**
Maternal education			
< High school	61 (18.5)	45 (21.4)	21 (13.8)
High school	56 (17.0)	36 (17.1)	25 (16.4)
Some college	73 (22.2)	46 (21.9)	34 (22.4)
Four year college degree	72 (21.9)	41 (19.5)	34 (22.4)
Post college education	67 (20.4)	42 (20.0)	38 (25.0)
Race/ethnicity			
Chinese	92 (28.0)	72 (34.3)*	41 (27.0)
Asian (non-Chinese)	21 (6.4)	13 (6.2)	9 (5.9)
Black	50 (15.2)	27 (12.8)	23 (15.1)
White	133 (40.4)	77 (36.7)	62 (40.8)
Other	33 (10.0)	21 (10.0)	17 (11.2)
Married/ living with partner	265 (80.6)	172 (81.9)	126 (82.9)
TONI-2 score	95.8 ± 11.4	95.8 ± 11.3	95.8 ± 13.0
Missing TONI	118 (35.9)	82 (39.0)	26 (17.1)**
Maternal exposure to ETS, reported as smoker in the home (%)	59 (17.9)	36 (17.1)	26 (17.1)
Ate fish during the pregnancy	233 (70.8)	150 (71.4)	110 (72.4)
Material hardship	31 (9.4)	20 (9.5)	16 (10.5)
Infant characteristics			
Birth weight (g)	3419.5 ± 469.1	3399.2 ± 472.5	3412.0 ± 487.4
Birth length (cm)	50.8 ± 2.8	50.5 ± 2.7*	50.6 ± 2.7
Birth head circumference (cm)	34.2 ± 1.5	34.2 ± 1.4	34.3 ± 1.5
Gestational age (days)	276.8 ± 9.9	276.4 ± 10.4	276.6 ± 9.5
Male	161 (48.9)	105 (50.0)	77 (50.7)
Proportion of first year breast-fed (% of 1 year)	0.24 ± 0.28	0.22 ± 0.27	0.26 ± 0.28
Residential characteristics			
Worked and/or lived within 1 mile of the WTC during any of the 4 weeks after 9/11	62 (18.8)	43 (20.5)	32 (21.0)
Worked and/or lived within 2 mile of the WTC during any of the 4 weeks after 9/11	141 (42.8)	94 (44.8)	73 (48.0)

Values are mean ± SD or *n* (%).

* Statistical comparison between those in the full cohort and those with cord blood measurements, *p* < 0.05.

** Statistical comparison between those in the full cohort and those in our study sample, *p* < 0.05.

**Table 3:** Association (95% CIs) between prenatal exposure to BDE-47 and indices of neurodevelopment at 12, 24, 36, 48, and 72 months of age.

					Model 2^b^	
	Age (months)	*n*	Univariate Change in score per ln-unit	Model 1^a^ Change in score per ln-unit	Change in score per ln-unit	Change in score per increase from the 25th to 75th percentile (IQR^c^)
MDI	12	118	−0.88 (−2.20 to 0.44)	−0.60 (−2.04 to 0.83)	−0.64 (−2.11 to 0.82)	−1.06 (−3.49 to 1.36)
	24	117	−2.88 (−5.20 to −0.55)*	−2.65 (−4.82 to −0.48)*	−3.12 (−5.25 to −0.99)*	−5.16 (−8.68 to −1.64)*
	36	114	−0.34 (−2.60 to 1.93)	−0.34 (−2.65 to 1.98)	−1.05 (−3.35 to 1.26)	−1.74 (−5.54 to 2.08)

PDI	12	118	−1.01 (−2.99 to 0.97)	−2.10 (−4.16 to −0.04)*	−2.09 (−4.20 to 0.03)	−3.46 (−6.95 to 0.05)
	24	115	−0.21 (−2.13 to 1.70)	−0.11 (−2.11 to 1.89)	−0.29 (−2.37 to 1.79)	−0.48 (−3.92 to 2.96)
	36	109	−1.52 (−4.13 to 1.08)	−1.64 (−4.52 to 1.23)	−1.81 (−4.69 to 1.07)	−2.99 (−7.76 to 1.77)

Full	48	104	−1.98 (−4.49 to 0.53)	−2.13 (−4.31 to 0.06)	−2.42 (−4.71 to −0.12)*	−4.00 (−7.79 to −0.20)*
	72	96	1.02 (−2.03 to 4.08)	−0.12 (−2.92 to 2.69)	−1.17 (−4.03 to 1.69)	−1.94 (−6.67 to 2.80)

Verbal	48	104	−1.41 (−4.00 to 1.19)	−2.16 (−4.60 to 0.27)	−2.75 (−5.28 to −0.22)*	−4.55 (−8.73 to −0.36)*
	72	96	2.31 (−0.94 to 5.57)	1.10 (−1.78 to 3.97)	0.09 (−2.82 to 3.01)	0.15 (−4.66 to 4.98)

Performance	48	104	−2.27 (−5.04 to 0.50)	−1.76 (−4.27 to 0.74)	−1.67 (−4.32 to 0.98)	−2.76 (−7.15 to 1.62)
	72	96	−0.54 (−3.54 to 2.46)	−1.27 (−4.22 to 1.68)	−2.14 (−5.20 to 0.93)	−3.54 (−8.60 to 1.54)

a Model 1 adjusted for age at testing, race/ethnicity, IQ of mother, sex of child, gestational age at birth, maternal age, ETS (yes/no), maternal education, material hardship, and breast-feeding.

b Model 2 adjusted for all covariates in model 1 plus language and location of interview.

c Interquartile range (IQR) for BDE-47 is 19.57 ng/g lipid.

* CIs do not include 0.00.

**Table 4:** Association (95% CIs) between prenatal exposure to BDE-99 and indices of neurodevelopment at 12, 24, 36, 48, and 72 months of age.

					Model 2^b^	
	Age (months)	*n*	Univariate Change in score per ln-unit	Model 1^a^ Change in score per ln-unit	Change in score per ln-unit	Change in score per increase from the 25th to 75th percentile (IQR^c^)
MDI	12	118	−0.56 (−1.82 to 0.71)	−0.47 (−1.85 to 0.92)	−0.52 (−1.92 to 0.88)	−0.83 (−3.08 to 1.41)
	24	117	−3.12 (−5.38 to −0.85)*	−2.67 (−4.78 to −0.56)*	−2.82 (−4.86 to −0.78)*	−4.52 (−7.79 to −1.25)*
	36	114	−0.63 (−2.83 to 1.57)	−0.80 (−3.03 to 1.43)	−1.09 (−3.29 to 1.11)	−1.75 (−5.27 to 1.78)

PDI	12	118	0.36 (−1.53 to 2.25)	−0.80 (−2.81 to 1.22)	−0.74 (−2.79 to 1.31)	−1.19 (−4.47 to 2.10)
	24	115	−0.33 (−2.19 to 1.54)	0.01 (−1.93 to 1.95)	−0.06 (−2.03 to 1.90)	−0.10 (−3.25 to 3.04)
	36	109	−0.66 (−3.18 to 1.86)	−0.71 (−3.47 to 2.05)	−0.89 (−3.62 to 1.84)	−1.43 (−5.80 to 2.95)

Full	48	104	−1.39 (−3.76 to 0.99)	−1.41 (−3.46 to 0.64)	−1.42 (−3.53 to 0.61)	−2.28 (−5.66 to 0.98)
	72	96	0.30 (−2.65 to 3.25)	−0.18 (−2.88 to 2.53)	−0.22 (−2.97 to 2.52)	−0.35 (−4.76 to 4.04)

Verbal	48	104	−1.11 (−3.55 to 1.34)	−1.72 (−3.99 to 0.55)	−1.88 (−4.16 to 0.40)	−3.01 (−6.67 to 0.64)
	72	96	0.99 (−2.18 to 4.16)	0.56 (−2.22 to 3.35)	0.76 (−2.02 to 3.54)	1.22 (−3.24 to 5.67)

Performance	48	104	−1.49 (−4.11 to 1.14)	−0.94 (−3.28 to 1.41)	−0.86 (−3.23 to 1.52)	−1.38 (−5.18 to 2.44)
	72	96	−0.54 (−3.44 to 2.35)	−0.89 (−3.74 to 1.96)	−1.20 (−4.16 to 1.75)	−1.92 (−6.67 to 2.80)

a Model 1 adjusted for age at testing, race/ethnicity, IQ of mother, sex of child, gestational age at birth, maternal age, ETS (yes/no), maternal education, material hardship, and breast-feeding.

b Model 2 adjusted for all covariates in model 1 plus language and location of interview.

c IQR for BDE-99 is 5.60 ng/g lipid.

* CIs do not include 0.00.

**Table 5:** Association (95% CIs) between prenatal exposure to BDE-100 and indices of neurodevelopment at 12, 24, 36, 48, and 72 months of age.

					Model 2^b^	
	Age (months)	*n*	Univariate Change in score per ln-unit	Model 1^a^ Change in score per ln-unit	Change in score per ln-unit	Change in score per increase from the 25th to 75th percentile (IQR^c^)
MDI	12	118	−0.99 (−2.39 to 0.41)	−0.62 (−2.16 to 0.92)	−0.72 (−2.29 to 0.85)	−1.00 (−3.17 to 1.18)
	24	117	−3.27 (−5.71 to −0.83)*	−2.95 (−5.33 to −0.56)*	−3.67 (−6.00 to −1.34)*	−5.09 (−8.32 to −1.86)*
	36	114	−0.56 (−2.97 to 1.85)	−1.04 (−3.55 to 1.47)	−1.89 (−4.36 to 0.58)	−2.62 (−6.04 to 0.80)

PDI	12	118	−0.72 (−2.83 to 1.38)	−1.98 (−4.20 to 0.24)	−1.93 (−4.21 to 0.35)	−2.68 (−5.84 to 0.49)
	24	115	−0.10 (−2.13 to 1.93)	0.26 (−1.97 to 2.49)	0.10 (−2.22 to 2.41)	0.14 (−3.08 to 3.34)
	36	109	−1.14 (−3.90 to 1.62)	−0.97 (−4.10 to 2.15)	−1.25 (−4.38 to 1.87)	−1.73 (−6.07 to 2.59)

Full	48	104	−3.29 (−5.95 to −0.63)*	−3.30 (−5.61 to −0.98)*	−3.68 (−6.05 to −1.28)*	−5.10 (−8.39 to −1.77)*
	72	96	−1.36 (−4.79 to 2.07)	−2.18 (−5.36 to 0.99)	−3.10 (−6.27 to 0.06)	−4.30 (−8.69 to 0.08)

Verbal	48	104	−2.29 (−5.06 to 0.48)	−2.87 (−5.49 to −0.25)*	−3.46 (−6.14 to −0.79)*	−4.80 (−8.51 to −1.10)*
	72	96	0.64 (−3.06 to 4.34)	−0.70 (−4.01 to 2.60)	−1.46 (−4.74 to 1.81)	−2.02 (−6.57 to 2.51)

Performance	48	104	−3.90 (−6.82 to −0.98)*	−3.32 (−5.98 to −0.66)*	−3.37 (−6.14 to −0.60)*	−4.67 (−8.51 to −0.83)*
	72	96	−3.20 (−6.51 to 0.11)	−3.12 (−6.45 to 0.21)	−4.02 (−7.41 to −0.63)*	−5.57 (−10.27 to −0.87)*

a Model 1 adjusted for age at testing, race/ethnicity, IQ of mother, sex of child, gestational age at birth, maternal age, ETS (yes/no), maternal education, material hardship, and breast-feeding.

b Model 2 adjusted for all covariates in model 1 plus language and location of interview.

c IQR for BDE-100 is 2.12 ng/g lipid.

* CIs do not include 0.00.

**Table 6:** Association (95% CIs) between prenatal exposure to BDE-153 and indices of neurodevelopment at 12, 24, 36, 48, and 72 months of age.

					Model 2^b^	
	Age (months)	*n*	Univariate Change in score per ln-unit	Model 1^a^ Change in score per ln-unit	Change in score per ln-unit	Change in score per increase from the 25th to 75th percentile (IQR^c^)
MDI	12	111	−0.24 (−1.74 to 1.26)	0.05 (−1.59 to 1.69)	0.02 (−1.65 to 1.68)	0.02 (−1.51 to 1.54)
	24	113	−0.42 (−2.90 to 2.06)	−1.26 (−3.68 to 1.17)	−1.71 (−4.07 to 0.65)	−1.57 (−3.73 to 0.60)
	36	107	1.78 (−0.54 to 4.09)	0.29 (−2.17 to 2.75)	−0.05 (−2.46 to 2.37)	−0.05 (−2.25 to 2.17)

PDI	12	111	−1.44 (−3.65 to 0.77)	−2.02 (−4.37 to 0.33)	−1.95 (−4.34 to 0.43)	−1.79 (−3.98 to 0.39)
	24	111	0.90 (−1.12 to 2.92)	1.20 (−1.03 to 3.44)	1.21 (−1.06 to 3.49)	1.11 (−0.97 to 3.20)
	36	102	−0.04 (−2.62 to 2.54)	0.04 (−2.96 to 3.05)	0.03 (−3.00 to 3.05)	0.03 (−2.75 to 2.79)

Full	48	97	−0.30 (−3.14 to 2.55)	−2.69 (−5.17 to −0.21)*	−3.09 (−5.64 to −0.54)*	−2.83 (−5.17 to −0.49)*
	72	92	0.02 (−3.28 to 3.32)	−3.40 (−6.57 to −0.22)*	−3.25 (−6.35 to −0.15)*	−2.98 (−5.82 to −0.14)*

Verbal	48	97	0.24 (−2.63 to 3.12)	−1.93 (−4.71 to 0.85)	−2.46 (−5.32 to 0.41)	−2.25 (−4.87 to 0.38)
	72	92	1.74 (−1.82 to 5.29)	−1.91 (−5.28 to 1.47)	−1.79 (−5.05 to 1.47)	−1.64 (−4.63 to 1.35)

Performance	48	97	−1.22 (−4.37 to 1.92)	−3.46 (−6.36 to −0.57)*	−3.62 (−6.62 to −0.63)*	−3.32 (−6.07 to −0.58)*
	72	92	−2.03 (−5.23 to 1.17)	−4.35 (−7.63 to −1.06)*	−4.19 (−7.48 to −0.89)*	−3.84 (−6.85 to −0.82)*

a Model 1 adjusted for age at testing, race/ethnicity, IQ of mother, sex of child, gestational age at birth, maternal age, ETS (yes/no), maternal education, material hardship, and breast-feeding.

b Model 2 adjusted for all covariates in model 1 plus language and location of interview.

c IQR for BDE-153 is 0.85 ng/g lipid.

* CIs do not include 0.00.
